# Are Fatigue and Pain Overlooked in Subjects with Stable Chronic Obstructive Pulmonary Disease?

**DOI:** 10.3390/diagnostics11112029

**Published:** 2021-11-02

**Authors:** Koichi Nishimura, Kazuhito Nakayasu, Mio Mori, Ryo Sanda, Ayumi Shibayama, Masaaki Kusunose

**Affiliations:** 1 Department of Respiratory Medicine, National Center for Geriatrics and Gerontology, Obu 474-8511, Japan; mio-mori@ncgg.go.jp (M.M.); ryo-sand@ncgg.go.jp (R.S.); kusunose@ncgg.go.jp (M.K.); 2 Data Research Section, Kondo Photo Process Co., Ltd., Osaka 543-0011, Japan; nakayasu@mydo-kond.co.jp; 3 Department of Nursing, National Center for Geriatrics and Gerontology, Obu 474-8511, Japan; ayuminarita3@ncgg.go.jp

**Keywords:** chronic obstructive pulmonary disease (COPD), the Brief Fatigue Inventory (BFI), the revised Short–Form McGill Pain Questionnaire 2 (SF-MPQ-2), the Kihon Checklist, fatigue, pain

## Abstract

Although there have been many published reports on fatigue and pain in patients with chronic obstructive pulmonary disease (COPD), it is considered that these symptoms are seldom, if ever, asked about during consultations in Japanese clinical practice. To bridge this gap between the literature and daily clinical experience, the authors attempted to gain a better understanding of fatigue and pain in Japanese subjects with COPD. The Brief Fatigue Inventory (BFI) to analyse and quantify the degree of fatigue, the revised Short–Form McGill Pain Questionnaire 2 (SF-MPQ-2) for measuring pain and the Kihon Checklist to judge whether a participant is frail and elderly were administered to 89 subjects with stable COPD. The median BFI and SF-MPQ-2 Total scores were 1.00 [IQR: 0.11–2.78] and 0.00 [IQR: 0.00–0.27], respectively. They were all skewed toward the milder end of the respective scales. A floor effect was noted in around a quarter on the BFI and over half on the SF-MPQ-2. The BFI scores were significantly different between groups regarding frailty determined by the Kihon Checklist but not between groups classified by the severity of airflow limitation. Compared to the literature, neither fatigue nor pain are considered to be frequent, important problems in a real-world Japanese clinical setting, especially among subjects with mild to moderate COPD. In addition, our results might suggest that fatigue is more closely related to frailty than COPD.

## 1. Introduction

Breathlessness is undoubtedly believed to be one of the most important perceptions experienced in subjects with chronic obstructive pulmonary disease (COPD). This is likely followed by coughing, as well as sputum production, symptoms which are described in most of the clinical practice guidelines [1]. Antoniu et al. has stated that the most prevalent, clinically significant extra-respiratory symptom was fatigue, which was reported in 95.7%, followed by pain in 74.5%, of patients hospitalized for a COPD exacerbation [2]. Upon closer examination of the published ranking lists of symptoms in subjects with stable COPD, Walke et al. reported that shortness of breath was followed by physical discomfort, fatigue, problems with appetite, anxiety, and pain [3]. Blinderman et al. found that lack of energy was in second place, while dry mouth was third, other pain (non-chest) was eighth, and chest pain twelfth [4]. Peters et al. also found that around half of patients with COPD had abnormal fatigue [5]. Guyatt et al. stipulated that one of the four domains should be named as fatigue during the development of the Chronic Respiratory Disease Questionnaire (CRQ), the first disease-specific tool for measuring quality of life globally [6]. Thus, although fatigue may be the second most frequent symptom that is reported after dyspnoea by subjects with COPD, it is a frequently ignored symptom in daily clinical practice [7].

Prevalence rates for pain have also been reported to be surprisingly high in subjects with COPD over the last decade [8,9,10,11,12,13,14,15,16,17]. In a systematic review published in 2015, Lee et al. reported that the pooled prevalence of pain in moderate to very severe COPD was 66% (95% CI, 44–85%) [18]. Despite these findings, we were unsure of their applicability to Japanese COPD patients as pain is seldom, if ever, asked about during consultations in this country; neither has it been included as a primary or secondary endpoint in most clinical trials. Notwithstanding, a recent review has stated that chronic pain warrants consideration within clinical practice guidelines for COPD [19].

When interviewing a candidate suspected of COPD, we often ask about breathlessness, cough, sputum and wheezing but not fatigue or pain. We are aware of only a small number of chest physicians in Japan who are of the opinion that these symptoms should be asked about in the consulting room. Although many published reports have studied fatigue and pain, it is unclear whether they should be checked during every examination. The aim of this study was to bridge the gap between the literature and daily clinical experience by gaining a better understanding of fatigue and pain in subjects with COPD. COPD is occasionally considered to be an accelerated aging disease since it is well known that aging of the lungs and COPD have many similarities and are sometimes difficult to distinguish from each other [20]. The frequency of fatigue, as well as breathlessness, is also thought to increase progressively with advancing years [21,22,23,24,25]. Since the age of patients with COPD is considered to be much older in Japan than in western countries [26], we hypothesized that aging could play a role in the appearance of symptoms such as fatigue and pain. Hence, the secondary purpose of the present study was to examine the prevalence of the above symptoms and their relationship with frailty in subjects with stable COPD [27,28].

## 2. Materials and Methods

### 2.1. Participants

We recruited 89 consecutive patients with stable COPD who attended the outpatient clinic at the Department of Respiratory Medicine of the National Center for Geriatrics and Gerontology (NCGG) from August 2018 to August 2020. The inclusion criteria were: (1) age more than 50 years; (2) smoking history exceeding 10 pack-years; (3) chronic fixed airflow limitation; (4) regular clinic attendance for more than half a year to avoid any changes induced by new medical interventions; (5) no uncontrolled co-morbidities and (6) no variation in treatment in the preceding four weeks. Chronic fixed airflow limitation was defined as a maximal ratio of forced expiratory volume in 1 s (FEV_1_) to forced vital capacity (FVC) of under 0.7. All participants gave written informed consent.

### 2.2. Measurements

Baseline measurements of the participants’ pulmonary function were taken in a single day. These comprised post-bronchodilator spirometry (CHESTAC-8800; Chest, Tokyo, Japan), residual volume (RV) using the closed-circuit helium method, and diffusing capacity for carbon monoxide (DL_CO_) measured by the single-breath technique as reported by the American Thoracic Society and European Respiratory Society Task Force in 2005 [29]. Calculations of the predicted values for FEV_1_ and vital capacity were performed as recommended by the Japan Respiratory Society [30].

### 2.3. Assessment of Fatigue, Pain, Breathlessness and Frailty

Validated Japanese versions of the following patient-reported outcome measurement tools were used in the present study; the Brief Fatigue Inventory (BFI) to analyse and quantify the degree of fatigue, the revised Short-Form McGill Pain Questionnaire (SF-MPQ-2) for measuring pain, the Dyspnoea-12 (D-12) to assess the severity of breathlessness and the Kihon Checklist to judge whether a participant is a frail elderly person. Participants were asked to complete these self-administered questionnaires under supervision in the aforementioned order (in a booklet form).

The BFI is a questionnaire originally designed to assess fatigue in cancer patients [31], but it has also been administered in subjects with COPD [13,32,33]. It consists of 9 numerical scales ranging from 0 to 10. The first three items in the BFI ask patients to rate the severity on an eleven-point rating scale with “0” being “no fatigue,” and “10” being “fatigue as bad as you can imagine.” An additional six items assess how greatly fatigue interferes with different aspects of daily activities. Each interference item is also scored on an eleven-point rating scale from “0” (does not interfere) to “10” (completely interferes). A mean BFI score is calculated as the mean of the intensity and interference items. The reliability of the Japanese version of the BFI was assessed by Okuyama et al. in the outpatient clinics of 6 oncology divisions and yielded a Cronbach’s alpha value of 0.96 [34]. Furthermore, using the same tool, they reported that fatigue severity should be categorized as mild (1–3), moderate (4–6), and severe (7–10).

Although the Short Form McGill Pain Questionnaire (SF-MPQ) has been reported to be successfully administered in subjects with stable or exacerbated COPD to quantify the degree of pain [15], it was revised to the SF-MPQ-2 by adding symptoms relevant to neuropathic pain and by modifying the response format. It has thus become a tool for measuring both neuropathic and non–neuropathic pain [35,36]. The SF-MPQ-2 comprises 22 items investigating 4 dimensions: 6 items in Continuous pain, 6 in Intermittent pain, 6 in Neuropathic pain and 4 in Affective descriptors. Each item has an eleven-point numerical rating scale from 0 to 10. A lower score indicates less severe pain. For each of dimensions, scores are calculated by taking the mean of the item ratings included in the scale. The total score is calculated to be the mean of all SF-MPQ-2 item ratings. The internal consistency, or Cronbach’s alpha coefficient, of the Japanese version of the SF-MPQ-2 has been reported to be 0.907 of the total score [36].

To assess the severity of dyspnoea, we used the D-12, which consists of twelve items (seven physical and five affective), each with a four-point grading scale (0–3), producing a Total Score (range 0–36, with higher scores representing more severe breathlessness) [37,38].

The authors also administered the Kihon Checklist to judge whether a participant is a frail elderly person [27,28,39,40]. This is a self-administered questionnaire comprising 25 items in a yes/no question format dealing with instrumental (3 items) and social activities of daily living (4 items), physical strength (5 items), nutritional status (2 items), oral function (3 items), cognitive status (3 items), and depression risk (5 items) [39,40]. One question concerns body mass index (BMI) and is usually self-scored but we calculated this using data collected at the same time as the pulmonary function tests. The Kihon Checklist total score, which is a sum of 25 answers, ranges from 0 (no frailty) to 25 (severe frailty) and patients’ frailty status was classified as robust (0–3), pre-frail (4–7) and frail (8–25), as reported in the literature [28,39,40].

### 2.4. Statistical Methods

Cronbach’s coefficient alpha was used to assess internal consistency. Score distributions of the tools were evaluated with the Shapiro-Wilk test and by inspection of histograms. Spearman’s rank correlation tests were used to examine relationships between two sets of data and differences between groups were assessed by the Steel-Dwass test. All *p* values less than 0.05 were deemed to be statistically significant. The results are expressed as mean ± standard deviation (SD) with some exceptions in the tables.

## 3. Results

### 3.1. Characteristics of the Study Subjects

A total of 89 consecutive patients (83 men) with COPD, and a wide range of FEV_1_ (69.8 ± 21.0%pred) were studied. Seventy-six subjects were former smokers while 13 were current smokers. Their demographic details, as well as the results of pulmonary function tests, are shown in Table 1. Using the classification of severity of airflow limitation of the Global Initiative for Chronic Obstructive Lung Disease (GOLD) criteria [1], 30 subjects (33.7%) were in GOLD 1 (defined as FEV_1_ ≥ 80% predicted), 46 (51.7%) in GOLD 2 (50% ≤ FEV_1_ < 80% predicted), 9 (10.1%) in GOLD 3 (30% ≤ FEV_1_ < 50% predicted) and 4 (4.5%) in GOLD 4 (FEV_1_ < 30% predicted).

### 3.2. Internal Consistency and Distribution of Scores

**Figure 1:**
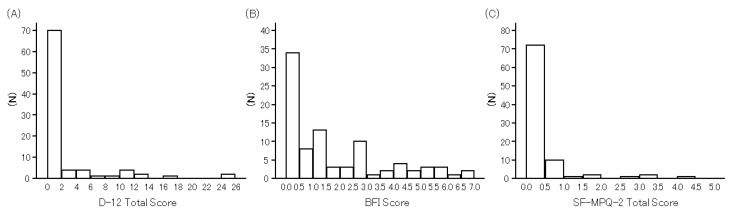
Frequency distribution histograms of the Dyspnoea-12 (D-12) Total Score (**A**), the Brief Fatigue Inventory (BFI) Score (**B**) and revised Short–Form McGill Pain Questionnaire 2 (SF-MPQ-2) Total Score (**C**) in 89 subjects with COPD. The D-12 is designed for measuring dyspnoea, BFI for fatigue and SF-MPQ-2 for pain. Higher scores in all the tools included herein indicate more severe impairment. The scores were all skewed toward the milder end of the respective scales.

The internal consistency of the BFI, SF-MPQ-2 Total, and D-12 Total scores as assessed by Cronbach’s coefficient alpha was excellent (alpha over 0.9) except for three subscales of the SF-MPQ-2, which ranged from 0.773 (both Continuous and Neuropathic pain) to 0.877 (Affective descriptors) (Table 2). Frequency distribution histograms of the scores obtained from the BFI, SF-MPQ-2 Total, and D-12 Total scores are shown in Figure 1 (Shapiro-Wilk test; *p* < 0.001, all). They were all skewed toward the milder end of the respective scales. A floor effect was noted in 22 subjects (24.7%) on the BFI, in 45 subjects (50.6%) on the SF-MPQ-2 and in 46 subjects (51.7%) on the D-12 Total (Table 2). According to Okayama’s proposal, the BFI score was 1.00 or more, that is more than mild fatigue, in 47 (52.8%) out of the 89 subjects.

### 3.3. Relationship between Fatigue and Physiological or Clinical Factors

Spearman’s rank correlation coefficients (Rs) between the BFI score and physiological or clinical factors are shown in Table 1. The BFI score may be marginally characterized by negative correlations with airflow limitation as well as diffusion capacity and by a positive association with hyperinflation (absolute value of Rs = 0.240 to 0.305). The Kihon Checklist Total score correlated most strongly with the BFI score (Rs = 0.531). In a comparison between groups stratified by airflow limitation severity, the BFI score was significantly different between GOLD1 and GOLD 3+4 (*p* < 0.05, Steel-Dwass test) but not between GOLD 1 and 2 nor between GOLD 2 and 3+4 (Table 3). As categorized by the Kihon Checklist total score, there were significant differences in the BFI scores between groups with and without frailty (Table 4).

### 3.4. Relationship between Pain and Physiological or Clinical Factors

There were no statistically significant correlations between the SF-MPQ-2 Total scores and clinical and physiological factors (Table 1). However, there was a significant correlation between the SF-MPQ-2 Total scores and both the Kihon Checklist Total and BFI scores (Rs = 0.293 and 0.233, respectively). There were no significant differences between the scores obtained for the SF-MPQ-2 Total and its four subscales and the GOLD criteria (Table 3). In addition, the SF-MPQ-2 Total, Neuropathic pain and Affective descriptors’ scores were significantly different between the robust and frail groups (Table 4).

### 3.5. Relationship between Dyspnoea and Physiological or Clinical Factors

The D-12 Total, Physical and Affect scores were significantly different between GOLD1 and GOLD3+4 and between GOLD2 and GOLD3+4 (Table 3), but there were no significant differences among the robust, pre-frail and frail groups (Table 4).

## 4. Discussion

Despite many published reports that fatigue and pain are frequently observed, important symptoms in subjects with stable COPD, our findings could provide little support for these assertions. Since the BFI and SF-MPQ-2 Total scores were remarkably skewed toward the milder end of the scales and high floor effects were observed, we could not clearly establish whether fatigue and pain were common problems in the participants. However, the findings deserve careful and thoughtful consideration since the D-12 scores were also skewed toward the milder end despite dyspnoea being considered one of the COPD-specific symptoms. Although the authors carefully selected validated Japanese versions of PRO measurement tools with a history of use in COPD [10,15,33,34,36], there is the possibility that neither the BFI nor the SF-MPQ-2 performed adequately in the present study. Another possible reason for the differences with previously reported findings may be the fact that there were considerable numbers of subjects with relatively mild to moderate COPD as mean FEV_1_ was 69.8 (21.0) %predicted. If more patients with severe COPD were enrolled, it is possible that the scores might have been more normally distributed.

The BFI scores were significantly different between groups as determined by frailty but not between groups classified by the severity of airflow limitation. This might suggest that fatigue is more closely related to frailty than COPD. Since it has been reported that fatigue is more frequently complained of in elderly people, frailty may lead to frequent development of fatigue in subjects with COPD as previously reported [21,22,24]. On the other hand, since dyspnoea was different between airflow limitation-based groups but not between frail and robust subjects, dyspnoea appears to be closely associated with COPD but only distantly with frailty.

To discuss the prevalence of fatigue as well as pain, standardized tools which have been psychometrically validated and reproducible should be used. Single, closed questions with a yes or no answer format are not recommended since they may lead to measurement errors such as overestimation of the specific symptoms. In addition to standardized instruments, reference scores for the general population or healthy non-smoking subjects are necessary to compare the prevalence between different groups. Although the authors performed a search for BFI reference scores, the knowledge and information obtained by preceding studies have been limited. Chen et al. first reported the reliability and validity of the BFI in subjects with COPD in 2016 [33]. They noted that the BFI score was 3.92 (2.51) at first administration and 3.66 (2.43) at the second in all COPD subjects. Furthermore, it was 3.53 (2.51) in 6 patients with mild COPD and there was no significant difference between groups classified by COPD severity. Chen et al. subsequently reported in 2018 that the BFI score was 4.3 (2.0) in subjects with COPD and that the prevalence of fatigue was 77% [13], but information on cut-off scores was not provided [41]. We found the prevalence of more than mild fatigue to be 52.8% but since this threshold of the BFI was originally developed by Okuyama et al. with results from cancer patients, our finding should be treated with caution [34]. The BFI score was 1.70 (1.92) across all subjects with COPD in the present study and we are unable to explain the wide variation in published findings. There is a wide range of reported prevalence of pain in the published literature. Maignan et al. reported that, in 50 subjects, the median SF-MPQ score (not SF-MPQ-2) was 29.7 [IQR: 13.6–38.2] at the AECOPD phase and 1.4 [0.0–11.2] at the stable phase, and 46 (92%) patients reported pain during AECOPD compared to 29 (58%) in the stable phase [15]. As in other reports, information on cut-off scores was not provided [15]. In the present study, the SF-MPQ-2 Total and subscale scores were likely to be very low in almost all subjects with COPD and the skewed distribution was quite pronounced. Although there have been some reports of concurrent pain and dyspnoea in subjects with COPD [13,42], the SF-MPQ-2 Total score was not significantly correlated with D-12 scores nor COPD severity in the present study. Therefore, we have formed the impression that chronic pain might be sporadically reported in some patients. We also note the possible role of coexisting illness although major comorbidity was excluded from the study.

Why do subjects with COPD experience fatigue or pain? Although an association between fatigue or pain and COPD has been frequently reported in the literature, only a few studies have explored the underlying mechanism of this association. It is considered that COPD is associated with systemic manifestations and comorbidities. One of the most important possible mechanisms is mediated by elevated levels of nonspecific inflammatory cytokines, which can derive from the lung and enter systemic circulation [43,44]. This ‘overspill’ hypothesis may include not only comorbidities such as skeletal muscle dysfunction, cardiovascular disease, osteoporosis, and diabetes but also fatigue and pain. The relationship with inflammatory markers requires further study. In addition, from the point of view of the ‘overspill’ hypothesis, comorbidities may have been one of the important outcomes. Since “no uncontrolled co-morbidities” was one of the inclusion criteria, most subjects with comorbidities were excluded from the present study. Data on comorbidities should have been collected in a quantitative way using the Charlson comorbidity index and this was a particular limitation of our study. Although it is reported that fibromyalgia should be distinguished from similar symptoms in subjects with severe asthma complaining of pain as well as extra-pulmonary asthma symptoms [45,46], it is believed that such patients were not included.

Some other limitations of the present study should be mentioned. First, frailty is considered to be one of the defining characteristics of aging and the developments of several concise measurement tools have consequently been reported in the literature. The Cardiovascular Health Study Index developed by Fried et al. has been the most widely used to assess this biological syndrome [27]. Other tools have also been validated, such as the Frailty Index and Clinical Frailty Scale using the cumulative deficit approach [47,48,49]. Although we used the Kihon Checklist to assess frailty status, variations in the classification of frailty between these screening tools might exist [50]. Second, there is the possibility of selection bias and care should be taken with any generalization of our results. Only patients who could regularly attend our outpatient clinic were recruited. Patients without any subjective symptoms and thus unaware of having COPD were not represented. Others who were unable to regularly attend our clinic due to the great physical effort involved would also have been excluded. This single-center study was also limited by its small sample size and the fact that most of the subjects were male, even though it includes most of the stable COPD patients who attended our hospital during the study period. The participants were overwhelmingly male because there were relatively few female COPD patients in Japan at the time. The study sample therefore reflects the reality of clinical COPD in our population.

## 5. Conclusions

In conclusion, the median BFI and SF-MPQ-2 Total scores were 1.00 [IQR: 0.11–2.78] and 0.00 [IQR: 0.00–0.27], respectively. They were all skewed toward the milder end of the respective scales. A floor effect was noted in around a quarter on the BFI and over half on the SF-MPQ-2. The BFI scores were significantly different between groups regarding frailty determined by the Kihon Checklist, but not between groups classified by the severity of airflow limitation. Compared to the literature, neither fatigue nor pain are considered to be frequent important problems in a real-world Japanese clinical setting, especially among subjects with mild to moderate COPD. In addition, our results might suggest that fatigue is more closely related to frailty than COPD.

## Figures and Tables

**Table 1:** Baseline characteristics in 89 subjects with COPD and Spearman’s rank correlation coefficients with Brief Fatigue Inventory (BFI) and Short-form McGill Pain Questionnaire (SF-MPQ)-2 Total scores.

			Median	IQR	Correlation Coefficients (Rs) with	
					BFI Score	SF-MPQ-2 Total Score
Age		years	78.0	74.0–82.0	0.165	0.162
BMI		kg/m^2^	22.6	20.9–24.4	0.036	0.121
Cumulative Smoking		pack-years	60.0	39.0–79.5	0.152	0.112
SVC		Liters	3.22	2.54–3.62	−0.247 *	0.089
FEV_1_		Liters	1.69	1.31–2.03	−0.305 **	0.062
FEV_1_/FVC		%	58.9	48.6–64.2	−0.242 *	0.015
RV/TLC ^(1)^		%	42.0	35.3–50.2	0.240 *	−0.039
DLco ^(2)^		mL/min/mmHg	10.86	6.83–13.55	−0.260 *	0.116
PaO_2_ ^(3)^		mmHg	78.1	71.9–86.6	−0.171	−0.088
Kihon Checklist Total score		(0–25)	5	2–9	0.531 ***	0.293 **
BFI score		(0–10)	1.00	0.11–2.78	N.A.	0.233 *
SF-MPQ-2 Total score		(0–10)	0.00	0.00–0.27	0.233 *	N.A.
	Continuous pain	(0–10)	0.00	0.00–0.00	0.226 *	0.753 ***
	Intermittent pain	(0–10)	0.00	0.00–0.00	0.165	0.600 ***
	Neuropathic pain	(0–10)	0.00	0.00–0.50	0.280 **	0.906 ***
	Affective descriptors	(0–10)	0.00	0.00–0.00	0.401 ***	0.556 ***
D-12 Total score		(0–36)	0	0–1	0.319 **	0.045
	D-12 Physical score	(0–21)	0	0–1	0.308 **	0.049
	D-12 Affect score	(0–15)	0	0–0	0.409 ***	−0.005

***: *p* < 0.001, **: *p* < 0.01, *: *p* ˂ 0.05, ^(1)^ *n* = 88, ^(2)^ *n* = 86, ^(3)^ two patients receiving oxygen; IQR, interquartile range; BFI, Brief Fatigue Inventory; SF-MPQ-2, Short-form McGill Pain Questionnaire 2; D-12, Dyspnoea-12. The numbers in parentheses denote possible score range.

**Table 2:** The internal consistency and the score distribution in the questionnaires.

Patient-Reported Outcomes		Possible Score Range	Items	Cronbach’s	Score Distribution							
			(*n*)	α Coefficient	Mean	SD	Median	Max	Min	IQR	Floor Effect	Ceiling Effect
BFI score		0–10	9	0.975	1.70	1.92	1.00	6.78	0.00	0.11–2.78	24.7%	0.0%
SF-MPQ-2 Total score		0–10	22	0.934	0.34	0.77	0.00	4.41	0.00	0.00–0.27	50.6%	0.0%
	Continuous pain	0–10	6	0.733	0.35	0.81	0.00	3.50	0.00	0.00–0.00	76.4%	0.0%
	Intermittent pain	0–10	6	0.906	0.23	0.75	0.00	5.00	0.00	0.00–0.00	83.1%	0.0%
	Neuropathic pain	0–10	6	0.733	0.49	0.91	0.00	4.33	0.00	0.00–0.50	56.2%	0.0%
	Affective descriptors	0–10	4	0.877	0.26	0.97	0.00	6.00	0.00	0.00–0.00	85.4%	0.0%
D-12 Total score		0–36	12	0.960	2.2	4.8	0	25	0	0–1	51.7%	0.0%
	D-12 Physical score	0–21	7	0.925	1.6	3.0	0	15	0	0–1	51.7%	0.0%
	D-12 Affect score	0–15	5	0.964	0.6	1.9	0	10	0	0–0	86.5%	0.0%

IQR, interquartile range; BFI, Brief Fatigue Inventory; SF-MPQ-2, Short-form McGill Pain Questionnaire 2; D-12, Dyspnoea-12.

**Table 3:** Comparison of clinical indices and scores obtained from fatigue, pain and dyspnoea.

			GOLD 1 (*n* = 30)				GOLD 2 (*n* = 46)				GOLD 3+4 (*n* = 13)			
			Median	IQR			Median	IQR			Median	IQR		
Age		years	77.0	71.0	—	82.0	78.0	74.0	—	84.0	77.0	73.0	—	80.0
BMI		kg/m^2^	22.9	21.1	—	24.2	22.9	21.0	—	25.1	21.8	18.3	—	23.4
Cumulative Smoking		pack-years	46.5 **	30.0	—	61.0	66.5	50.0	—	86.0	50.0	40.0	—	75.0
SVC		Liters	3.50 **	3.22	—	4.05	3.08 ^§^	2.44	—	3.57	2.39 ^¶¶¶^	2.14	—	2.61
FEV_1_		Liters	2.18 ***	1.98	—	2.45	1.59 ^§§§^	1.39	—	1.83	0.85 ^¶¶¶^	0.68	—	1.03
FEV_1_/FVC		%	64.3 ***	59.8	—	66.5	56.8 ^§§§^	48.8	—	62.5	35.2 ^¶¶¶^	33.1	—	40.8
RV/TLC ^(1)^		%	35.4 **	32.4	—	43.5	43.4 ^§^	37.6	—	49.3	52.4 ^¶¶¶^	50.2	—	55.9
DLco ^(2)^		mL/min/mmHg	12.34	9.52	—	14.51	10.25	6.72	—	13.54	8.38 ^¶^	5.94	—	10.59
PaO_2_ ^(3)^		mmHg	79.0	76.0	—	87.8	80.4 ^§§^	72.4	—	87.7	70.9 ^¶¶¶^	64.8	—	72.3
Kihon Checklist Total score		(0–25)	3	1	—	6	6	2	—	10	6	3	—	15
BFI score		(0–10)	0.44	0.00	—	1.89	1.00	0.00	—	2.78	2.89 ^¶^	1.00	—	5.67
SF-MPQ-2 Total score		(0–10)	0.02	0.00	—	0.45	0.05	0.00	—	0.27	0.00	0.00	—	0.23
	Continuous pain	(0–10)	0.00	0.00	—	0.50	0.00	0.00	—	0.00	0.00	0.00	—	0.00
	Intermittent pain	(0–10)	0.00	0.00	—	0.00	0.00	0.00	—	0.00	0.00	0.00	—	0.00
	Neuropathic pain	(0–10)	0.00	0.00	—	0.50	0.00	0.00	—	0.67	0.00	0.00	—	0.33
	Affective descriptors	(0–10)	0.00	0.00	—	0.00	0.00	0.00	—	0.00	0.00	0.00	—	0.00
D-12 Total score		(0–36)	0	0	—	1	1 ^§§^	0	—	1	8 ^¶¶^	1	—	12
	D-12 Physical score	(0–21)	0	0	—	1	1 ^§§^	0	—	1	7 ^¶¶^	1	—	8
	D-12 Affect score	(0–15)	0	0	—	0	0 ^§§^	0	—	0	0 ^¶¶^	0	—	5

In comparison between GOLD1 and GOLD2 (Steel-Dwass test), ***: *p* < 0.001, **: *p* < 0.01. In comparison between GOLD2 and GOLD3+4 (Steel-Dwass test), ^§§§:^ *p* < 0.001, ^§§^: *p* < 0.01, ^§^: *p* ˂ 0.05. In comparison between GOLD1 and GOLD3+4 (Steel-Dwass test), ^¶¶¶^: *p* < 0.001, ^¶¶^: *p* < 0.01, ^¶^: *p* ˂ 0.05, ^(1)^ *n* = 88, ^(2)^ *n* = 86, ^(3)^ two patients receiving oxygen. IQR, interquartile range; BFI, Brief Fatigue Inventory; SF-MPQ-2, Short-form McGill Pain Questionnaire 2; D-12, Dyspnoea-12. The numbers in parentheses denote possible score range.

**Table 4:** Comparison of clinical indices and scores obtained from fatigue, pain and dyspnoea measurement tools between robust, pre-frail and frail groups classified by the Kihon Checklist in 89 subjects with COPD.

			Robust (*n* = 37)				Pre-Frail (*n* = 23)				Frail (*n* = 29)			
			Median	IQR			Median	IQR			Median	IQR		
Age		years	75.0	69.0	—	80.0	77.0	75.0	—	82.0	79.0 ^¶^	76.0	—	85.0
BMI		kg/m^2^	22.7	21.1	—	24.2	21.7	19.6	—	24.2	23.0	21.1	—	24.8
Cumulative Smoking		pack-years	50.0	37.0	—	61.0	56.0	38.0	—	80.0	71.8 ^¶^	50.0	—	80.0
SVC		Liters	3.39	3.00	—	3.75	3.03	2.47	—	3.33	2.77 ^¶^	2.32	—	3.57
FEV_1_		Liters	2.01	1.51	—	2.30	1.67	1.39	—	1.99	1.49 ^¶¶^	1.03	—	1.87
FEV_1_/FVC		%	63.0	53.8	—	65.0	58.5	46.7	—	64.6	57.5	42.5	—	62.5
RV/TLC ^(1)^		%	38.9 *	33.1	—	45.0	45.2	37.0	—	51.0	47.0 ^¶^	36.5	—	52.4
DLco ^(2)^		mL/min/mmHg	12.92 **	10.93	—	15.01	8.85	6.34	—	11.97	9.52 ^¶¶^	5.04	—	12.55
PaO_2_ ^(3)^		mmHg	79.0	75.2	—	88.0	81.3 ^§^	72.8	—	92.8	73.5 ^¶^	69.4	—	79.5
Kihon Checklist Total score		(0–25)	2 ***	1	—	2	5 ^§§§^	5	—	6	11 ^¶¶¶^	9	—	15
BFI score		(0–10)	0.22 *	0.00	—	1.00	1.89 ^§^	0.11	—	2.78	2.89 ^¶¶¶^	1.11	—	5.44
SF-MPQ-2 Total score		(0–10)	0.00	0.00	—	0.14	0.05	0.00	—	0.23	0.23 ^¶^	0.00	—	0.59
	Continuous pain	(0–10)	0.00	0.00	—	0.00	0.00	0.00	—	0.00	0.00	0.00	—	0.83
	Intermittent pain	(0–10)	0.00	0.00	—	0.00	0.00	0.00	—	0.17	0.00	0.00	—	0.00
	Neuropathic pain	(0–10)	0.00	0.00	—	0.17	0.00	0.00	—	0.83	0.33 ^¶^	0.00	—	1.33
	Affective descriptors	(0–10)	0.00	0.00	—	0.00	0.00	0.00	—	0.00	0.00 ^¶¶^	0.00	—	0.75
D-12 Total score		(0–36)	0	0	—	1	1	0	—	1	1	0	—	5
	D-12 Physical score	(0–21)	0	0	—	1	1	0	—	1	1	0	—	5
	D-12 Affect score	(0–15)	0	0	—	0	0	0	—	0	0	0	—	0

In comparison between robust and pre-frail (Steel-Dwass test), ***: *p* < 0.001, **: *p* < 0.01, *: *p* ˂ 0.05. In comparison between pre-frail and frail (Steel-Dwass test), ^§§§^: *p* < 0.001, ^§^: *p* ˂ 0.05. In comparison between robust and frail (Steel-Dwass test), ^¶¶¶^: *p* < 0.001, ^¶¶^: *p* < 0.01, ^¶^: *p* ˂ 0.05. ^(1)^ *n* = 88, ^(2)^ *n* = 86, ^(3)^ two patients receiving oxygen. IQR, interquartile range; BFI, Brief Fatigue Inventory; SF-MPQ-2, Short-form McGill Pain Questionnaire 2; D-12, Dyspnoea-12. The numbers in parentheses denote possible score range.

## Data Availability

The datasets generated during and/or analyzed during the current study are available from the corresponding author on reasonable request.
